# Impact of pemetrexed chemotherapy on the gut microbiota and intestinal inflammation of patient-lung-derived tumor xenograft (PDX) mouse models

**DOI:** 10.1038/s41598-020-65792-6

**Published:** 2020-06-04

**Authors:** Cindy Pensec, Florence Gillaizeau, Dominique Guenot, Anne Bessard, Thomas Carton, Sébastien Leuillet, Mario Campone, Michel Neunlist, Hervé M. Blottière, Françoise Le Vacon

**Affiliations:** 1 Biofortis Mérieux NutriSciences, 44800 Saint Herblain, France; 2 IMODI Consortium:, http://www.imodi-cancer.org/; 3 Université de Strasbourg (Unistra), EA 3430, U1113 IRFAC, Fédération de Médecine Translationnelle de Strasbourg (FMTS), 67200 Strasbourg, France; 4 TENS, INSERM U1235, Institut des Maladies de l’Appareil Digestif du CHU de Nantes, Université de Nantes, 44035 Nantes, France; 5 Institut de Cancérologie de l’Ouest, Nantes, France; 6 Micalis Institute, INRA, AgroParisTech, Université Paris-Saclay, 78350 Jouy-en-Josas, France; 7 MetaGenoPolis, INRA, Université Paris-Saclay, 78350 Jouy-en-Josas, France

**Keywords:** Non-small-cell lung cancer, Metagenomics

## Abstract

Chemotherapy remains the gold standard for advanced cancer. Pemetrexed, a chemotherapeutic agent used in non-small cell lung cancer, can induce significant side effects in patients. Although microbiota’s role in the efficacy and/or toxicity of chemotherapy agents has been demonstrated, the impacts of pemetrexed on the gut microbiota and on gastrointestinal inflammation remain unknown. The objective of this study was to evaluate the impact of pemetrexed and the tumor graft on the gut microbiota composition in immunodeficient mice. The faecal microbiota composition was studied with metabarcoding before, 24-h and one week after treatment. The colon epithelial barrier integrity was evaluated by histological examination, intestinal permeability measurement, and selected cytokines quantification. The tumor graft induced some variations in the microbiota composition. Pemetrexed further increased the relative abundance of *Enterobacteriaceae* and 3 families from the Firmicutes phylum: *Enterococcaceae*, *Lactobacillaceae* and *Streptococcaceae*. Pemetrexed also significantly altered the epithelial barrier integrity, which was associated with early inflammation. This pilot study shows that the association of a lung tumor graft with pemetrexed causes an alteration in the microbiota composition. Such information increases our knowledge about the impact of chemotherapy on the microbiota, which could help to minimize side effects and improve therapeutic effectiveness in the future.

## Introduction

Lung cancer remains the most frequent cancer in the world, both in terms of incidence (1.8 million new cases/year) and mortality (1.6 million deaths/year)^1^. During the last decade, great progress has been made in the theranostics of lung cancer based on personalized pharmacotherapy, particularly for the most common subtype, non-small cell lung carcinoma (NSCLC). In addition to histological classifications, tumor screening is now performed to obtain a molecular profile and to examine some predictive and prognostic biomarkers. Thanks to major advancements in our knowledge, major driver mutations have been well described and can be inhibited with targeted therapies; for instance, tyrosine kinase inhibitors or monoclonal antibodies can inhibit angiogenesis or the Epidermal Growth Factor pathway. More recently, the development of immunotherapy allowed for the development of new strategies in lung cancer^2,3^. However, for the majority of lung cancers, chemotherapy remains the treatment of choice because most patients present at diagnosis, with locally advanced or metastatic cancer^4^. Pemetrexed is one of the recommended drugs, combined with cisplatin or carboplatin as a first-line treatment for advanced NSCLC, but is also used as a maintenance therapy and second- and third-line therapy^3^.

Pemetrexed is a multi-target anti-folate drug that inhibits several enzymes involved in the folate pathway: thymidylate synthase, dihydrofolate reductase, and glycinamide ribonucleotide formyltransferase^5,6^. These enzymes are involved in purine and pyrimidine nucleotide metabolism for DNA and RNA synthesis^3^. Given that pemetrexed has broad-spectrum activity, this chemotherapeutic agent induces toxicity such as neutropenia, skin rashes, diarrhoea, mucositis, and nausea/vomiting^4,5^.

The role of the gut microbiota in carcinogenesis has been recently revealed as complex, but it is now well known that the gut microbiota can contribute to an increased risk of cancer and can participate in progression^7^. Some bacteria can promote the initiation and progression of cancer *via* different processes. Microbiota alterations can favour opportunistic pathogens and can contribute to higher mucosal permeability, resulting in bacterial or bacterial product translocation; as a result, components of both the innate and adaptive immune systems can be activated, leading to chronic inflammation. Translocated bacterial products, such as toxins or metabolites, can affect cell cycle regulation, cell proliferation, and DNA integrity and can influence cancer development and progression^7,8^. In addition, recent studies have demonstrated the important role of the microbiota in modulating the efficacy and toxicity of chemotherapies, and more recently, of immunotherapies^7^. Indeed, the antitumor efficacy could be modulated by bacteria through their influence on the host immune response. For instance, the effect of cyclophosphamide was reduced in germ-free mice and in mice with depleted Gram-positive bacteria following antibiotic treatment^9^, but the presence of *Lactobacillus johnsonii* and *Enterococcus hirae* can restore the efficacy of cyclophosphamide. One of the side effects of this chemotherapy is alterations in the gut mucosa, along with the translocation of intraluminal bacteria into secondary lymphoid organs. The translocation of *L. johnsonii* and *E. hirae* could promote the antitumor adaptive immune response by increasing the intratumoral CD8 + T cell/T regulatory cell ratio and by activating pathogenic T helper 17 cells and memory Th1 cell immune responses^7^. In the case of irinotecan treatment, the gut microbiota increases its toxicity. In fact, bacterial β-glucuronidase uses the glucuronide of the inactive form of the molecule as a carbon source. The molecule are consequently reactivated in cytotoxic form causing intestinal toxicity and diarrhoea^10^.

The relationship between pemetrexed and the gut microbiota has not yet been studied, although pemetrexed is a routine drug used for lung cancer treatment. We therefore decided to investigate the impact of pemetrexed on the gut microbiota composition to highlight a potential dysbiosis (imbalance of gut microbiota) and to evaluate the effects of pemetrexed on the colon epithelial barrier integrity and inflammation. Our study used a model based on ectopic patient-derived xenografts (PDXs) developed from human lung tumors.

## Methods

### Animals and ethical considerations

Thirty-nine healthy female CB17 SCID (severe combined immunodeficient) mice (six- to eight-weeks-old) were obtained from Charles River (L’Arbresles, France) and maintained in specific pathogen-free (SPF) conditions in accordance with the Federation of European Laboratory Animal Science Association (FELASA) guidelines^11^. Animal housing and experimental procedures were conducted according to the French and European Regulations and the NRC Guide for the Care and Use of Laboratory Animals. The protocol was approved by the Oncodesign animal care and use ethical committee (Oncomet), which is certified by the French authorities (CNREEA agreement #91). The tumor sample was obtained from a xenograft tumor bank that was previously established^12^.

### Experimental study design

**Figure 1:**
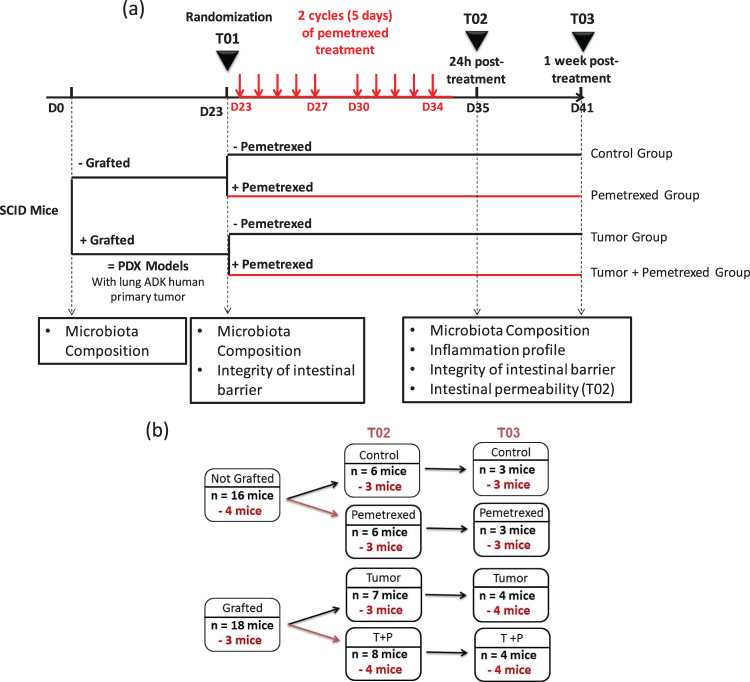
Study Design (**a**) Experimental study design and collection time points. (**b**) Groups of mice for the experiment. The numbers in black correspond to the mice that underwent microbiota analysis. The numbers in red correspond to the mice that were sacrificed for RT-qPCR and histology. The number of mice in black includes the 3 or 4 sacrificed mice. T + P, mice bearing a tumor and treated with pemetrexed. (ADK = Adenocarcinoma; PDX = Patient-derived xenograft).

The study design is presented in Fig. 1. After primary amplification in five healthy female CB17 SCID mice, the xenografted human lung adenocarcinoma tissue was divided into 30- to 50-mg fragments that were subcutaneously implanted into the right flank of 18 mice, while 16 mice remained graft-free (day 0 of the study). Twenty-three days later (denoted as time point T01), when the tumor volume had reached 150 to 250 mm^3^, 34 mice were randomized into one of the four groups: “Control” (C group – no tumor and no treatment), “Tumor” (T group – tumor and no treatment), “Pemetrexed” (P group – no tumor and treatment), and “Tumor + Pemetrexed” (P + T group – tumor and treatment) groups. Mice treated with pemetrexed (ALIMTA, Eli Lilly and Company, Indianapolis, USA) received two cycles of once daily intraperitoneal injections (75 mg/kg in NaCl 0.9%) for 5 consecutive days for 2 weeks. Mice were treated from day 23 (D23) to D27 and then from D30 to D34 (Fig. 1). All mice were weighed twice a week, and the tumor volume was measured with callipers.

### Faecal sample collection

Faeces were collected prior to treatment (T01), 24-h after treatment (T02), and one week (T03) after the end of the 2 pemetrexed cycles. These samples were stored at −80 °C until microbiota composition analysis.

### 16S metabarcoding analysis

DNA from PDX mouse stools was extracted after bead-beating using Maxwell® 16 Tissue DNA Purification Kits (Promega, Charbonnière-les-Bains, France) following the manufacturer’s instructions. DNA extraction was performed on 100-mg faecal samples. Double-stranded DNA concentrations were measured by fluorimetry using a Qubit® 3.0 Fluorometer, Qubit® dsDNA broad range, and a high sensitivity assay for the least concentrated DNA samples (Invitrogen Thermo Fisher Scientific, Illkirch-Graffenstaden, France). DNA samples were stored at −20 °C until further processing for microbiota analysis and bacterial quantification by QPCR.

The V3-V4 region of the bacterial 16 S ribosomal RNA (16 S rRNA) gene was amplified from microbiota genomic DNA using universal primers: 341 F (5′-CCTACGGGNGGCWGCAG-3′) and 785 R (5′-GACTACHVGGGTATCTAATCC-3′), as previously described^13^. PCR was performed by using 5 ng/µL of DNA, 0.2 µM of primers, and 1X KAPA HiFi HotStart Ready Mix (Roche, Boulogne-Billancourt, France) in a final volume of 25 µL. PCR cycling was performed with an initial denaturation at 95 °C for 5 min, followed by 25 cycles at 98 °C for 30 s, annealing at 55 °C for 30 s, elongation at 72 °C for 30 s, and a final extension of 72 °C for 5 min. The amplicons were purified using AMPure XP beads (Agencourt, Beckman Coulter, Villepinte, France) according to the manufacturer’s instructions and were analysed by a Bioanalyzer on DNA chips from Diversilab (Agilent Technologies, Les Ulis, France). After DNA quantification, a library was generated using a Nextera XT Index kit (Illumina, Paris, France). Each library was purified with an Agencourt AMPure kit (Beckman Coulter, Villepinte, France), quality controlled on a 2100 Bioanalyzer platform (Agilent Technologies, Les Ulis, France), and quantified on a Qubit® 2.0 fluorometer (Life Technologies, Thermo Fisher Scientific, Illkirch-Graffenstaden, France) using a Qubit® dsDNA BR Assay Kit (Life Technologies, Thermo Fisher Scientific). Then, all libraries were normalized to 4 nM, pooled and denatured with 0.2 N NaOH, diluted to 6 pM and mixed with 20% 6 pM denatured phiX, according to an Illumina protocol (Part # 15044223revB). The amplicons were sequenced using an Illumina MiSeq platform using a 2 × 250 paired-end MiSeq kit V2 (Illumina, Paris, France).

The sequences generated from faecal samples were analysed using an in-house bioinformatic pipeline adapted from the MOTHUR software^14^. Briefly, sequences were trimmed and aligned to the V3-V4 region of the 16 S gene of the Greengenes database, which was formatted by MOTHUR (gg_13_5_99 release). Chimera sequences were removed using the UCHIME algorithm. Reads were classified using a naive Bayesian classifier against Silva Release 123 and were formatted for MOTHUR with a bootstrap cutoff of 70%. Sequences were then clustered into operational taxonomic units (OTUs) using furthest-neighbour clustering with a similarity threshold of 97%. For each sample, the OTU-based microbial diversity was estimated by calculating the Shannon and rarefied Chao1 (to 12650 reads) indices with the R package phyloseq (R version 3.4.3)^15^.

### qPCR of total bacteria

The total bacterial quantity was established by PCR using primers from the 16 S region: Uni331modF 5′-TCCTACGGGAGGCAGCAGTG-3′ and E533modR 5′-TTACCGCGGCTGCTGGCACG-3′^16^. DNA from samples (5 ng/µL) was amplified with primers at 0.7 µM and 1X SYBR qPCR Premix Ex Taq (Takara, Saint-Germain-en-Laye, France) in a final volume of 25 µL. PCRs included the following conditions: 95 °C for 3 min; followed by 40 cycles of 95 °C for 15 s, 60 °C for 30 s, 72 °C for 30 s; and a final hold at 4 °C, using a 7500 Real Time PCR System (Applied Biosystems).

### Intestinal permeability *in vivo*

Intestinal permeability was measured as described previously^17^. FITC (fluorescein isothiocyanate)-dextran (4 kDa, Sigma-Aldrich, Saint-Quentin Fallavier, France) was administered by gavage at a concentration of 600 mg/kg body weight (10 mL/kg of a 60 mg/mL solution). Four hours later, the animals were anaesthetized with isoflurane gas (1.0–1.5% Forane®, Abbott, France). Blood was collected by cardiac puncture and was centrifuged for 10 min at 2,000 g and +4 °C, and the plasma was stored at −80 °C. Plasma was diluted in PBS (1/9 volume). Plasma FITC levels were determined by a fluorescence spectrophotometer (Varioskan, Thermo Fisher Scientific) with an excitation wavelength of 485 nm and an emission wavelength of 528 nm. The readings were analysed with the Skan It software (Thermo Fisher Scientific).

### RNA extraction

Total RNA from proximal and distal colon was extracted by using NucleoSpin RNA/protein (Macherey Nagel, Düre, Germany) following the manufacturer’s instructions. Briefly, 30 mg of tissue, RA1 lysis buffer, and β-mercaptoethanol were homogenized in lysing Matrix D tubes with Precellys-24 (6800 tr/min, 2 cycles of 25 s) (Bertin Technologies, Montigny-le Bretonneux, France). The cellular lysate was filtered with a Nucleospin Filter, and ethanol was added to separate RNA and proteins. The RNA was eluted from the column with ethanol and was collected in RNase-free water. A treatment with TURBO™ DNAase (Thermo Fisher Scientific) was carried out.

### Cytokine mRNA expression analysis with RT-qPCR

cDNA was synthesized from 1 µg of total RNA using RT superscript III (Invitrogen, Thermo Fisher Scientific). Reverse transcriptase PCR was performed by a StepOnePlus System with SYBR Green Master Mix for the gene expression of TNFα (UP GAACTTCGGGGTGATCGGTC,NM_001278601,1 and LP GCCACTCCAGCTGCTCCTCC NM_013693,3), IL-10 (UP GACTTTAAGGGTTACTTGGGTTGC, NM_010548.2 and LP AGAAATCGATGACAGCGCCTC), and IL-1β (UP GCCTCGTGCTGTCGGACCCATA NM_008361.4 and LP TTGAGGCCCAAGGCCACAGGT) or with a TaqMan probe (Thermo Fisher Scientific) for IL-6 (Mm00446190_m1) and MCP1 (Mm00441242_m1). All genes were normalized to the S6 housekeeping gene. This gene encodes the ribosomal protein S6, a component of the 40 S ribosomal subunit involved in regulating translation.

### Histology

Colons were collected for histology when mice were sacrificed at T02. The colon was washed with a 0.9% NaCl solution, cut longitudinally and rolled up to obtain a swiss roll, which was fixed in 4% formalin and embedded in paraffin (Histosec®, Merck, Darmstadt, Germany). Histological tissue sections and eosin-haematoxylin (H&E) staining were performed on a MicroPICell platform (University of Nantes, France). After H&E staining, each microscopic tissue section was scanned with a Nanozoomer (Hamamatsu Photonics, Japan). The analyze of the crypt length, the crypt width, the number of Goblet cells per crypt and the mesure of the score of leukocyte infiltration were performed on the NDP View software (Hamamatsu Photonics, Japan). The score of leukocyte infiltration ranges from 0 (no infiltration) to 4 (very high infiltration).

### Statistical analyses

The relative variations in body weight and tumor volume from T01; the relative abundance of each taxon at the phylum, family and genus levels; the Shannon index (a marker of both diversity and evenness in microbiota composition); and the rarefied Chao1 index were studied using analysis of variance (ANOVA) for repeated measurements. Two-way ANOVA considered the group, the time, and their interaction. For body weight and tumor volume, the p-values were adjusted by Tukey’s method for pairwise comparisons between groups at each time point. For the analysis of the relative abundances of taxa, only taxa present in average in all samples at a threshold ≥0.05% or present in at least 10% of samples at a threshold ≥0.05% were analysed (statistically compared). A Benjamini-Hochberg procedure was used to control the false discovery rate (FDR) due to multiple hypothesis testing (adjusted p-values are presented). The procedure was used at each taxonomic classification level for the main effects of the model (multiple hypothesis tests on all taxa) and at each taxon level for the between-group comparisons (pairwise comparisons between groups at each time) and within-group comparisons (pairwise comparisons between times for each group). Intestinal permeability, inflammatory cytokine mRNA expression, crypt length, crypt width, the number of goblet cells, the scores of inflammatory cell infiltration and the total bacteria number were compared between the four groups using the Kruskal-Wallis test. Post hoc pairwise comparisons were conducted with Dunn’s test. The results are presented as the observed means ± the standard errors of the mean (SEM) or the estimated means with a 95% confidence interval (95% CI). A p-value < 0.05 was considered statistically significant. Inferential statistics were performed using SAS^®^ software version 9.3 (SAS Institute Inc., Cary, NC, USA) or GraphPad Prism 7 (GraphPad Software, San Diego, CA). Graphical representations were generated using R software version 3.3.2 (GG Plot 2) for microbiota data and GraphPad Prism 7 for other data.

## Results

### Pemetrexed transiently impacted the overall mouse weight and had a mild effect on tumor volume

**Figure 2:**
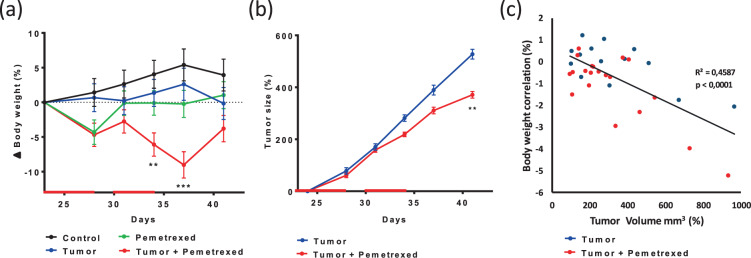
Pemetrexed impacted the mouse weight and tumor volume. (**a**) Percentage body weight changes (mean ± SEM) of the mice, normalized to D23 (start of treatment), over the 41-day study period. Groups were compared with ANOVA for repeated measurements, followed by Tukey’s test at each time point. Only significant results from the comparison of the “Tumor + Pemetrexed” VS “Control” groups are shown (two mice in the T group were sacrificed at D34 for ethical reasons) (**b**) Tumor volume of over the 41-day study period mice normalized to D23 (beginning of the treatment) (mean ± SEM). Groups were compared with ANOVA for repeated measurements. (**c**) Correlation between body weight changes and tumor volume normalized at the end of each treatment (D28 and D34). Statistical significance: **p < 0.01; ***p < 0.001.

At the end of the first pemetrexed cycle (at D27), only the groups P and P + T had a significant lost of weight (Fig. 2a). The mean relative weight loss (from D23 *i.e*., before starting the treatment) was estimated to be −4.30% [95% CI: −7.87%; −0.74%] in the P group (*p* = 0.0191), −4.69% [95% CI: −8.07%; −1.31%] in the T + P group (*p* = 0.0076), and 1.43% [95% CI: −2.62%; 5.47%] in the C group (*p* = 0.4817, which is not significant compared to the P and T + P groups, *p* = 0.1488 and *p* = 0.0975, respectively). During the two days between the two cycles (D28–29), mice in the P group returned to their before-treatment weight (−0.13% [95% CI: −3.69%; 3.44%]), while mice in the P + T group returned close to their initial weight (−2.75% [95% CI: −6.13%; 0.63%]). After the end of the second treatment cycle (at D34), the difference between the two groups was more important than during the first cycle and was close to statistical significance (adjusted *p* = 0.0740). Mice in the P group had a mean weight comparable to that at the start of the treatment (at D23; −0.10% [95% CI: −9.48%; −2.71%]), whereas the relative loss of weight in the P + T group was estimated to be −6.09% [95% CI: −9.48%; −2.71%]. Throughout the experiment, mice from the C and T groups progressively gained weight, except 2 mice in the T group that rapidly lost weight with important tumor growth at D34. These mice were sacrificed at T02, and therefore, did not further impact the body weight of their group.

The efficacy of pemetrexed was evaluated between mice with and without treatment by examining the changes in tumor volume over time. As shown in Fig. 2b, a significant reduction in tumor volume was observed following pemetrexed administration. At the end of the experiment (D41), the mean tumor volume was 527.4 mm^3^ [450.87; 603.87] in the T group and 371.1 mm^3^ [307.87; 434.25] in the T + P group, corresponding to a moderate, yet significant effect of pemetrexed on tumor growth (−156.30 mm^3^ [−255.52; 57.08], *p* = 0.0028). Interestingly, 24-h after each treatment cycle (D28 and D35), there was a moderate, negative linear correlation between the body weight change and tumor volume, regardless of the group (Fig. 2c, Pearson correlation coefficient ρ = −0.68, *p* < 0.001).

### Tumor engraftment modified the microbiota composition

**Figure 3:**
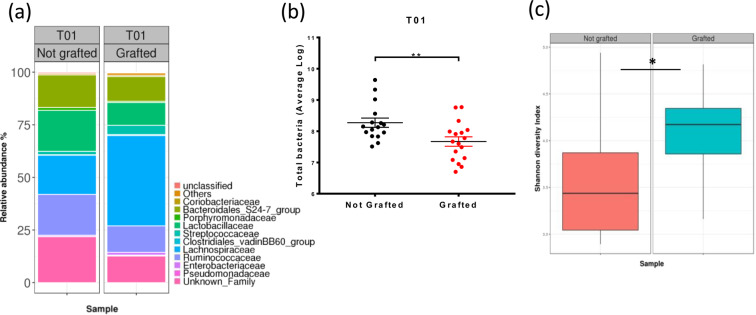
Impact of the tumor on the microbiota composition. (**a**) Microbiota composition at the family level at T01 in control (Not grafted, n = 16) and Grafted (n = 18) mouse groups, as assessed by 16 S rDNA metabarcoding. Families present at a threshold ≥0.5% on average in all samples or present in at least 10% of samples at a threshold ≥0.5% are shown. (**b**) Quantity of total bacteria ± SEM per group at T01, as measured by the qPCR of the 16 S rDNA gene. (**c**) Microbiota diversity established with the Shannon index at T01. Statistical significance: *p < 0.05; **p < 0.01.

The presence of a tumor led to several modifications in the microbiota composition (Fig. 3a and Supplemental Table 1). The phylum that was most impacted by the tumor graft was Firmicutes. Among the most abundant families, *Lachnospiraceae* and *Streptococcaceae* significantly increased in response to tumor grafts, whereas *Ruminococcaceae* and *Clostridiaceae_1* significantly decreased. Microbiota composition perturbations were associated with a significant decrease in the total quantity of bacteria in the DNA extract of faeces from grafted mice (8.3 log of the copy number ± 0.15 for control mice and 7.7 log ± 0.15 for grafted mice, *p* = 0.0076) (Fig. 3b). In addition, the microbiota diversity increased following tumor engraftment at T01 (D23). This increase in diversity was supported by the Shannon index (3.581 ± 0.142 for control mice *versus* 4.054 ± 0.138 for grafted mice; *p* = 0.0232) (Fig. 3c).

### Pemetrexed induced some dysbiosis in faecal microbiota

**Figure 4:**
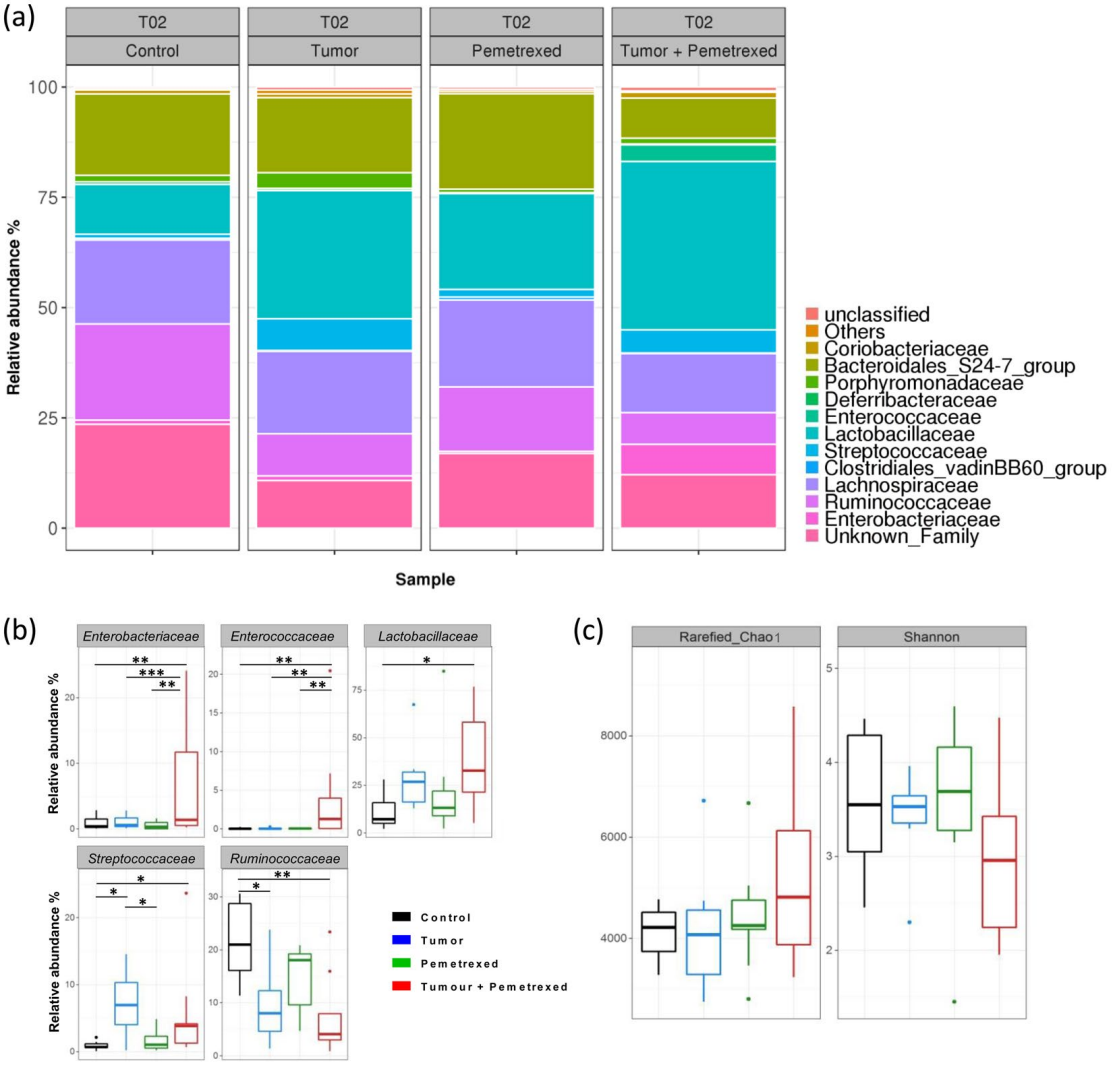
Impact of pemetrexed on the microbiota composition. (**a**) Microbiota composition at the family level at T02 in the 4 groups, as assessed by 16 S rRNA metabarcoding. (**b**) Relative abundance of the five most impacted families per group in each group. Groups were compared at T02 with ANOVA for repeated measurements with an FDR adjustment for multiple comparisons. Number of mice in the Control group = 6; Pemetrexed group = 6, Tumor group = 7 and Tumor + Pemetrexed = 8. (**c**) Microbiota diversity and richness established with the Shannon and rarefied Chao1 at T02. Statistical significance: *p < 0.05; **p < 0.01; ***p < 0.001.

The gut microbiota composition in the different groups of mice was analysed by a 16 S metabarcoding approach at T02 to study the impact of pemetrexed 24-h after the end of treatment (D35; Fig. 4). Pemetrexed treatment in grafted mice caused several microbiota perturbations compared to control mice (Fig. 4a). Indeed, for the Proteobacteria phylum, the *Enterobacteriaceae* family was significantly more abundant in the T + P group than in the three other groups (Fig. 4a,b, Supplemental Table 2, *p* = 0.0013, *p* = 0.0013, and *p* = 0.0004 for the T, C and P groups, respectively).

In addition, three families from the Firmicutes phylum (*Enterococcaceae, Lactobacillaceae* and *Streptococcaceae*) were significantly more abundant in response to tumor and/or treatment (Fig. 4a,b, Supplement Table 2). The *Enterococcaceae* family was significantly more abundant in the T + P group than in the three other groups (*p* = 0.0020 with the three groups). The *Lactobacillaceae* family was also significantly more abundant in the T + P group (38.17% ± 5.64% in the T + P group, *p* = 0.0193 compared with the C group). Moreover, in the absence of treatment, the mean relative abundance of the *Lactobacillaceae* family was greater in mice with tumors (T group) than in mice without tumors (C group), but the difference did not reach statistical significance (29.06% ± 6.75% in the T group compared to 11.33% ± 6.75% in the C group, *p* = 0.1346). Similar significant increases were observed for *Streptococcaceae* in groups T and T + P (5.24% ± 1.2% in the T + P group and 7.18% ± 1.44% in the T group compared to 0.91% ± 1.2% in the C group; respectively *p* = 0.0475 and *p* = 0.0174).

By contrast, the *Ruminococcaceae* family (Firmicutes phylum) was significantly less abundant in response to tumor and/or treatment (7.177% ± 2.45% in the T + P group and 9.6% ± 2.92% in the T group compared to 21.80% ± 2.92% in the C group; respectively *p* = 0.0016 and *p* = 0.0124). (Fig. 4a,b).

Interestingly, the differences between groups with and without tumor were already statistically significant for *Streptococcaceae* and *Ruminococcaceae* before starting treatment (T01) (Fig. 3a and Supplemental Table 2).

These results were supported by the lower Shannon index in the T + P group than in the other groups (Fig. 4c). An increase in the microbiota richness was also observed for the rarefied Chao1 index. There was no significant difference in the total bacteria number in faeces among the groups (data not shown).

One week after the end of pemetrexed treatment, the gut microbiota composition was studied to examine whether the observed dysbiosis was maintained. Overall, the gut microbiota returned to a normal composition at T03 (data not shown), and the increase in *Enterobacteriaceae* was no longer present in the T + P group. However, the decrease in *Ruminococcaceae* was still present and was more severe than at T02, as the mean relative abundance in T + P was 3.2% ± 3.2 compared to 16.3% ± 3.9 (*p* = 0.0344) in the C group. A significant increase in *Lactobacillaceae*, which was already present at T02, was still observed at T03 (64.1% ± 7.3 in the T + P group compared to 27.1% ± 9.0 in the control group; *p* = 0.0060).

### Pemetrexed weakly impacted the gut inflammatory status

**Figure 5:**
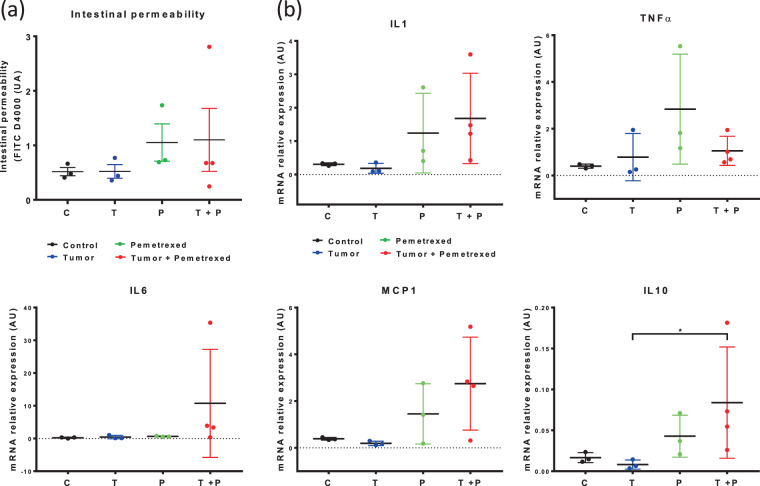
Impact of pemetrexed on intestinal permeability and inflammation. (**a**) Intestinal permeability was evaluated based on the amount of FITC-dextran found in the plasma in the 4 groups at T02. (**b**) mRNA expression of inflammatory cytokines in the proximal colon, as measured by RT-qPCR at T02. The four groups are the Control (C, N = 6), Tumor (T, N = 6), Pemetrexed (P, N = 7), and Tumor + Pemetrexed (T + P, N = 8). Data were analysed with the Kruskal-Wallis test and were corrected with Dunn’s test. *p < 0.05.

The intestinal permeability was measured at T02 (D35) by examining the presence of FITC-dextran in the plasma (Fig. 5a). The observed mean values of intestinal permeability were greater in the T + P (1.101 UA) and P (1.052 UA) groups than in the other groups, but the difference among the groups was not significant (*p* = 0.3851 and *p* = 0.0747, respectively, compared to the C group, Fig. 5a). Among the 5 selected cytokines (IL-1β, IL-6, IL-10, TNFα, and MCP1), the mRNA expression of IL-10 was significantly higher in the T + P group than in the T group in proximal colon (*p* = 0.0280, Fig. 5b); a smaller non-significant effect was observed in the P group compared to the T group (*p* = 0.3193). In distal colon, no significant results are observed (data not shown). In addition, IL-1β and MCP1 mRNA expression was higher in the T + P and P than in the other groups. At T03, the expression of all cytokines returned to the baseline values (data not shown).

### Pemetrexed modified the integrity of the colon epithelial barrier

**Figure 6:**
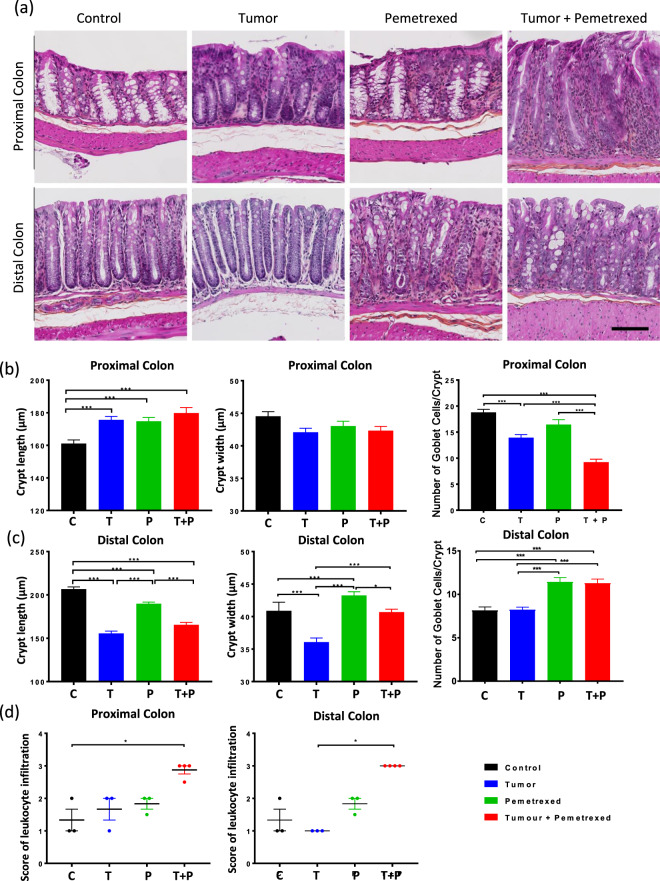
Impact of pemetrexed on the colon epithelial barrier integrity. (**a**). Haematoxylin-eosin staining of the proximal and distal colon 24-h post-treatment (T02) of the 4 groups (scale = 100 µm). (b and c) Crypt length, width and the number of goblet cells in the proximal (**b**) and distal (**c**) colon. (**d**) Score of inflammatory cell infiltration at T02 in the proximal and distal colon. The graphs show the mean ± SEM of each group. Data were analysed with the Kruskal-Wallis test and were corrected with Dunn’s test. Control (C, N = 6), Tumor (T, N = 6), Pemetrexed (P, N = 7), and Tumor + Pemetrexed (T + P, N = 8). Statistical significance: *p < 0.05; **p < 0.01; ***p < 0.001.

In the proximal colon, the crypt length was significantly greater in the T, P, and T + P groups than in the C group (*p* < 0.0001, *p* = 0.0003, and *p* = 0.0007, respectively, Fig. 6a,b), whereas the crypt width was not significantly different among the groups. The number of goblet cells was significantly lower in the T and T + P groups than in C group (*p* < 0.0001 and *p* < 0.0001, respectively).

In opposite, in the distal colon, significant changes were observed for crypt length (decrease in groups T, P and T + P compared to group C (*p* = 0.0003, *p* < 0.0001 and *p* < 0.0001, respectively, Fig. 6c). The mean crypt width was lowest in group T and was highest in group P, and these two groups were significantly different from group C (*p* < 0.001 and *p* = 0.0005, respectively). The number of goblet cells in the distal colon was significantly higher in the P and T + P groups than in the C group (*p* < 0.0001 and *p* < 0.0001, respectively) and T group (*p* < 0.0001 and < 0.001, respectively).

In both the proximal and distal colon, the infiltration of inflammatory cells was significantly higher in the T + P group (score 3) than in the C and T groups respectively (*p* = 0.0367, *p* = 0.0161, Fig. 6d).

## Discussion

In the present study, we analysed the impact of pemetrexed on the gut microbiota composition, the colon mucosal integrity and inflammation in a mouse xenograft model of a human lung adenocarcinoma. The tumor graft induced variations in faecal microbiota composition, whereas pemetrexed treatment significantly altered the epithelial barrier integrity and was associated with early inflammation.

This observation of the impact of the tumor on the microbiota composition before treatment allowed us to document that the perturbation in the microbiota composition may be due in part to the tumor itself and not only to chemotherapy. In our study, the presence of a grafted tumor significantly increased the microbiota diversity but decreased the total number of bacteria per gram of faeces and slightly changed the microbiota composition; indeed, we observed several bacterial families impacted by the presence of a tumor as *Lachnospiraceae, Clostridiaceae*, *Ruminococcaceae* and *Streptococcaceae*. *Lachnospiraceae* and *Ruminococcaceae* are known to contain butyrate-producing species^18,19^. These families produce large amounts of short-chain fatty acids (SCFAs), especially acetate and butyrate, the latter being the major energy source for colonic epithelial cells^19^. The paradox in our study was that grafted tumors induced both an increase in *Lachnospiraceae* and a decrease in *Ruminococcaceae*. The low abundance of *Ruminococcaceae* may lead to a decrease in butyrate production, and several studies have shown that butyrate can affect the host immune response and that a lack of luminal butyrate induced nutritional deficiency in the colonic epithelium^20,21^. In fact, in the gut microbiota of CRC patients, an important part of the structural imbalance is a significant depletion of butyrate-producing bacteria^22–24^. Wang *et al*., show that the Lachnospiraceae family were less abundant^22^. However, in our results, the abundance of Lachnospiraceae increases in presence of the tumor. This discrepancy between our findings and previous studies might be due to multiple factors including diet, species, gender, age and the scheme of drug used. However, increased abundance of Lachnospiriaceae has still been reported in chronic Inflammatory Bowel Disease (IBD)^25^, Irritable Bowel Syndrome with Diarrhea (IBS-D)^26^, obesity^27,28^ and after stress^29^. The increase of *Streptococcaceae* was also significant following tumor grafting. The higher presence of this family has been associated with metabolic syndrome and colon cancer^30^.

The second part of this study examined the impact of pemetrexed treatment, with or without tumors, on tumor growth and the gut microbiota composition. First, our study demonstrated that pemetrexed induced significant weight loss in mice, indicating that this drug has potential toxic effects. This effect appeared to be strengthened by the presence of a tumor, as shown by the second pemetrexed cycle. Indeed, only the mice from the P + T group lost a significant amount of weight, in contrast to the P group. Moreover, a significant correlation between weight loss and tumor volume was established. The more the weight decreased, the more the tumor grew, suggesting that the efficacy of pemetrexed may have decreased because of its toxicity. Pemetrexed has a broad spectrum of antitumor activity and causes considerable toxicity in patients^5^. Myelosuppression is the major toxic effect that is encountered^31^, and grade 3–4 neutropenia with gastrointestinal toxicity occurs in approximately 50% of patients^32^. Interestingly, we observed that weight loss was ameliorated several days after the end of pemetrexed treatment, supporting the involvement of pemetrexed in this effect.

Concerning the impact of pemetrexed on the gut microbiota composition, a significant increase in two families, *Enterobacteriaceae* and *Enterococcaceae*, was only observed in the T + P group. This suggests that the association of the tumor and treatment might lead to the expansion of these families. These bacteria are frequently regarded as opportunistic pathogens. For example, the increase of *Enterococcaceae*, a family including pro-inflammatory opportunistic pathogens, has been seen in faecal samples from colorectal cancer patients^33^ and is commonly associated with metabolic disorders^34^. The significant increase in *Enterobacteriaceae* was the most striking observation in the microbiota perturbations in our mice, and this increase has also been reported in human patients suffering from metabolic disorders, obesity, IBD, diabetes and cancer^34–36^. In mice, Hughes *et al*. proposed that intestinal inflammation could reduce the ability of colonocytes to perform β-oxidation, causing an increase in oxygen in the gut lumen and promoting the proliferation of facultative anaerobic bacteria, such as *Enterobacteriaceae*^37^.

Interestingly, we also observed a decrease in *Ruminococcaceae* in the T group, which was amplified in the T + P group. As this decrease was observed after tumor grafting, but not in tumor-free animals, we assume that the loss of *Ruminococcaceae* was induced by the tumor rather than by the treatment.

To evaluate the resilience of gut microbiota after pemetrexed treatment, we analysed the gut microbiota composition one week after the end of the treatment (at D41, T03). Interestingly, the increase in *Enterobacteriaceae* was completely abolished. However, some variations persisted, such as the decrease in *Ruminococcaceae* and the increase in *Lactobacillaceae*, possibly due to the growth of the tumor. In fact, *Ruminococcaceae* was already observed at T01, before pemetrexed treatment. Concerning *Lactobacillaceae*, no significant increase was observed at T01 (after the tumor graft), but a trend could be observed.

Thus, the association of the tumor and pemetrexed appeared to amplify dysbiosis. In the future, it would be interesting to perform whole-metagenome shotgun sequencing to study the potential microbial metabolic functions involved in the pemetrexed-associated dysbiosis profile in PDX mice.

The importance of the symbiotic relationship between intestinal bacteria and epithelial cells has been well established, especially for the homeostatic functions of the intestine^38^. Based on the effects of pemetrexed, we hypothesize that (i) pemetrexed causes a dysbiosis that impacts the integrity of the epithelial barrier through the degradation of the mucus layer by certain gut bacteria. Indeed, the mucus layer can be an attachment site for bacteria and in addition, mucins are important carbon sources for some bacterial species^39^. (ii) Pemetrexed can act directly on the epithelial barrier, causing alterations in the cellular structure, and then on the mucus layer, which promotes the disturbance of the microbiota. Indeed, pemetrexed inhibits cell replication through the inhibition of three enzymes involved in DNA synthesis^38^. As the intestinal epithelium is one of the most rapidly dividing tissues^38^, it is highly probable that pemetrexed causes direct damage to intestinal epithelial cells. In our histological study, the combination of T + P had a more deleterious impact on epithelial integrity than was seen in the other groups. Additionally, alterations in crypt integrity, the decrease in goblet cell number, and the increased intestinal permeability in the proximal colon may have altered mucus production. These changes are associated with an increase in IL-10 production and inflammatory cell infiltration in the T + P group. A more permeable epithelium may facilitate bacterial or bacterial-product translocation, causing inflammation, as shown by the increased production of cytokines. In fact, the mucus barrier helps to maintain the mutualistic relationship between host immunity and bacteria and reduces leukocyte activation^40^. The disruption of this barrier may explain the early inflammation features observed in the proximal colon. It should be noted that SCID mice are severely but not completely deficient in functional B and T lymphocytes^33^; thus, the inflammatory response might have been less important than that in immunocompetent mice.

Interestingly, in our experiments, all histologic and inflammation perturbations observed 24-h after pemetrexed treatment disappeared one week after the end of treatment. A similar reversal has been described after a short course of antibiotic treatment^41^. Indeed, the microbiota has the capacity to resist alterations and to return to its original symbiosis between the host and microbiota composition^42^.

## Conclusions

This pilot study supports the hypothesis that tumor engraftment in association with chemotherapy, here pemetrexed, can disrupt the microbiota balance and can impact colon barrier integrity. Our results need to be confirmed in other PDX models to deepen our understanding of the relationship between the microbiota, cancer and inflammation following pemetrexed treatment. Finally, it will be essential to increase our knowledge about the impact of chemotherapy on the gut microbiota to establish strategies to minimize the intestinal side effects of these drugs. One of the major questions will be whether the bacterial dysbiosis or the barrier disruption occurs first.

### Ethics approval

All animal experiments were performed in accordance with the French and European Regulations and the NRC Guide for the Care and Use of Laboratory Animals. The protocol was approved by the Oncodesign animal care and use ethical committee (Oncomet), which is certified by the French authorities (CNREEA agreement #91).

## Supplementary information

Supplemental Tables 1 and 2.

## Data Availability

All data generated and analysed during this study are included in this manuscript and in the supplementary information files. Further details are available from the corresponding author upon request.
