# Supplementary material for: Impact of pemetrexed chemotherapy on the gut microbiota and intestinal inflammation of patient-lung-derived tumor xenograft (PDX) mouse models
Source: Sci Rep. 2020 Jun 4;10:9094. doi: 10.1038/s41598-020-65792-6 (PMC7272463; doi:10.1038/s41598-020-65792-6)
Supplement: Supplementary file 1 — Supplemental Tables 1 and 2. [file 41598_2020_65792_MOESM1_ESM.pdf]

Article Title: IMPACT OF PEMETREXED CHEMOTHERAPY ON THE GUT MICROBIOTA AND INTESTINAL INFLAMMATION OF PATIENT-LUNG-DERIVED TUMOUR XENOGRAFT (PDX) MOUSE MODELS

Author names: Cindy Pensec[1,2], Florence Gillaizeau[1], Dominique Guenot[2,3], Anne Bessard[4], Thomas Carton[1,2], Sébastien Leuillet[1,2], Mario Campone[5], Michel Neunlist[4], Hervé M. Blottière[6,7] and Françoise Le Vacon[1,2*].

Supplemental Table 1: Relative abundance of families present in the two groups at T01 (LSmeans (SEM)). Groups were compared at T01 for study the impact of tumor graft in control group and grafted group with ANOVA for repeated measurements with a FDR adjustment for multiple comparisons. Statistical significance: * *p*<0.05; ** *p*<0.01; *** *p*<0.001.

Phylum	Family	Time Point	Group	Lsmeans (SEM)	FDR p-value
Actinobacteria	Cellulomonadaceae	T01	Not Grafted	0.029 (0.035)	0.0325
		T01	Grafted	0.158 (0.034)	
	Coriobacteriaceae	T01	Not Grafted	0.105 (0.116)	0.0325
		T01	Grafted	0.584 (0.113)	
Bacteroidetes	Bacteroidales_S24-7_group	T01	Not Grafted	15.482 (2.617)	0.3337
		T01	Grafted	11.78 (2.538)	
	Porphyromonadaceae	T01	Not Grafted	1.354 (0.492)	0.2946
		T01	Grafted	0.578 (0.477)	
Deferribacteres	Deferribacteraceae	T01	Not Grafted	0.13 (0.082)	0.8479
		T01	Grafted	0.152 (0.08)	
Firmicutes	Bacillaceae	T01	Not Grafted	0.032 (0.04)	0.0451
		T01	Grafted	0.16 (0.039)	
	Clostridiaceae_1	T01	Not Grafted	0.098 (0.026)	0.0451
		T01	Grafted	0.015 (0.025)	
	Clostridiales_vadinBB60_group	T01	Not Grafted	0.222 (0.115)	0.1626
		T01	Grafted	0.475 (0.111)	
	Erysipelotrichaceae	T01	Not Grafted	0.191 (0.043)	0.2451
		T01	Grafted	0.115 (0.041)	
	Lachnospiraceae	T01	Not Grafted	18.625 (3.457)	0.0004
		T01	Grafted	42.918 (3.353)	
	Lactobacillaceae	T01	Not Grafted	19.476 (3.194)	0.0860

		T01	Grafted	10.841 (3.099)	
	Paenibacillaceae	T01	Not Grafted	0.013 (0.016)	0.0325
		T01	Grafted	0.069 (0.016)	
	Ruminococcaceae	T01	Not Grafted	19.405 (1.91)	0.0325
		T01	Grafted	12.577 (1.853)	
	Streptococcaceae	T01	Not Grafted	1.622 (0.673)	0.0325
		T01	Grafted	4.376 (0.653)	
Proteobacteria	Caulobacteraceae	T01	Not Grafted	0.037 (0.038)	0.0325
		T01	Grafted	0.185 (0.037)	
	Comamonadaceae	T01	Not Grafted	0.006 (0.009)	0.0325
		T01	Grafted	0.038 (0.009)	
	Enterobacteriaceae	T01	Not Grafted	0.571 (0.344)	0.1772
		T01	Grafted	1.29 (0.334)	
	Oxalobacteraceae	T01	Not Grafted	0.01 (0.014)	0.0325
		T01	Grafted	0.061 (0.013)	
	Pseudomonadaceae	T01	Not Grafted	0.091 (0.087)	0.0325
		T01	Grafted	0.441 (0.084)	
Saccharibacteria	Unknown_Family	T01	Not Grafted	21.859 (2.638)	0.0325
		T01	Grafted	12.67 (2.559)	
-	unclassified	T01	Not Grafted	0.576 (0.057)	0.0325
		T01	Grafted	0.365 (0.055)	

Supplemental Table 2: Relative abundance of families present in the four groups at T02 (LSmeans (SEM)). Groups were compared at T02 with ANOVA for repeated measurements with a FDR adjustment for multiple comparisons. G01 = Tumor, G02 = Tumor + Pemetrexed, G03 = Control and G04 = Pemetrexed. Statistical significance: * p<0.05; ** p<0.01; *** p<0.001.

Phylum	Family	Time-point	Group T - LSmeans (SEM)	Group T+P - LSmeans (SEM)	Group C - LSmeans (SEM)	Group P - LSmeans (SEM)	Comparison	FDR adj. p value
Actinobacteria	Cellulomonadaceae	T02	0.067 (0.039)	0.026 (0.033)	0.015 (0.039)	0.037 (0.035)	G01 vs G02	0.8349
		T02	.	.	.	.	G01 vs G03	0.8349
		T02	.	.	.	.	G01 vs G04	0.8349
		T02	.	.	.	.	G02 vs G03	0.8349
		T02	.	.	.	.	G02 vs G04	0.8349
		T02	.	.	.	.	G03 vs G04	0.8349
	Coriobacteriaceae	T02	0.838 (0.348)	1.341 (0.291)	0.909 (0.348)	0.558 (0.307)	G01 vs G02	0.6576
		T02	.	.	.	.	G01 vs G03	0.8865

		T02	.	.	.	.	G01 vs G04	0.6576
		T02	.	.	.	.	G02 vs G03	0.6576
		T02	.	.	.	.	G02 vs G04	0.4113
		T02	.	.	.	.	G03 vs G04	0.6576
Bacteroidetes	Bacteroidales_S24-7_group	T02	17.018 (4.678)	9.072 (3.914)	18.449 (4.678)	21.646 (4.126)	G01 vs G02	0.3934
		T02	.	.	.	.	G01 vs G03	0.8293
		T02	.	.	.	.	G01 vs G04	0.6907
		T02	.	.	.	.	G02 vs G03	0.3854
		T02	.	.	.	.	G02 vs G04	0.1808
		T02	.	.	.	.	G03 vs G04	0.7318
	Porphyromonadaceae	T02	3.561 (1.106)	1.309 (0.925)	1.481 (1.106)	0.768 (0.975)	G01 vs G02	0.3676
		T02	.	.	.	.	G01 vs G03	0.3751
		T02	.	.	.	.	G01 vs G04	0.3676
		T02	.	.	.	.	G02 vs G03	0.9054
		T02	.	.	.	.	G02 vs G04	0.8262
		T02	.	.	.	.	G03 vs G04	0.8262
Deferribacteres	Deferribacteraceae	T02	0.456 (0.179)	0.202 (0.149)	0.537 (0.179)	0.203 (0.158)	G01 vs G02	0.4383
		T02	.	.	.	.	G01 vs G03	0.9000
		T02	.	.	.	.	G01 vs G04	0.4383
		T02	.	.	.	.	G02 vs G03	0.4383
		T02	.	.	.	.	G02 vs G04	0.9967
		T02	.	.	.	.	G03 vs G04	0.4383
Firmicutes	Bacillaceae	T02	0.054 (0.038)	0.029 (0.032)	0.021 (0.038)	0.031 (0.034)	G01 vs G02	0.9691
		T02	.	.	.	.	G01 vs G03	0.9691
		T02	.	.	.	.	G01 vs G04	0.9691
		T02	.	.	.	.	G02 vs G03	0.9691
		T02	.	.	.	.	G02 vs G04	0.9691
		T02	.	.	.	.	G03 vs G04	0.9691
	Clostridiaceae_1	T02	0.010 (0.034)	0.016 (0.029)	0.017 (0.034)	0.083 (0.030)	G01 vs G02	0.9949
		T02	.	.	.	.	G01 vs G03	0.9949
		T02	.	.	.	.	G01 vs G04	0.2968
		T02	.	.	.	.	G02 vs G03	0.9949
		T02	.	.	.	.	G02 vs G04	0.2968
		T02	.	.	.	.	G03 vs G04	0.2968

	Clostridiales_vadinBB60_group	T02	0.196 (0.151)	0.136 (0.126)	0.391 (0.151)	0.649 (0.133)	G01 vs G02	0.7632
		T02	.	.	.	.	G01 vs G03	0.4338
		T02	.	.	.	.	G01 vs G04	0.0806
		T02	.	.	.	.	G02 vs G03	0.3042
		T02	.	.	.	.	G02 vs G04	0.0389 (*)
		T02	.	.	.	.	G03 vs G04	0.3042
	Enterococcaceae	T02	0.043 (0.833)	3.767 (0.697)	0.014 (0.833)	0.023 (0.735)	G01 vs G02	0.0020 (**)
		T02	.	.	.	.	G01 vs G03	0.9936
		T02	.	.	.	.	G01 vs G04	0.9936
		T02	.	.	.	.	G02 vs G03	0.0020 (**)
		T02	.	.	.	.	G02 vs G04	0.0020 (**)
		T02	.	.	.	.	G03 vs G04	0.9936
	Erysipelotrichaceae	T02	0.265 (0.063)	0.028 (0.053)	0.098 (0.063)	0.030 (0.055)	G01 vs G02	0.0197 (*)
		T02	.	.	.	.	G01 vs G03	0.1274
		T02	.	.	.	.	G01 vs G04	0.0197 (*)
		T02	.	.	.	.	G02 vs G03	0.5081
		T02	.	.	.	.	G02 vs G04	0.9800
		T02	.	.	.	.	G03 vs G04	0.5081
	Lachnospiraceae	T02	18.678 (4.596)	13.389 (3.845)	19.031 (4.596)	19.714 (4.053)	G01 vs G02	0.7606
		T02	.	.	.	.	G01 vs G03	0.9569
		T02	.	.	.	.	G01 vs G04	0.9569
		T02	.	.	.	.	G02 vs G03	0.7606
		T02	.	.	.	.	G02 vs G04	0.7606
		T02	.	.	.	.	G03 vs G04	0.9569
	Lactobacillaceae	T02	29.061 (6.747)	38.170 (5.645)	11.331 (6.747)	21.712 (5.951)	G01 vs G02	0.3648
		T02	.	.	.	.	G01 vs G03	0.1346
		T02	.	.	.	.	G01 vs G04	0.4167
		T02	.	.	.	.	G02 vs G03	0.0193 (*)
		T02	.	.	.	.	G02 vs G04	0.1346
		T02	.	.	.	.	G03 vs G04	0.3648
	Paenibacillaceae	T02	0.037 (0.019)	0.020 (0.016)	0.009 (0.019)	0.026 (0.017)	G01 vs G02	0.7964
		T02	.	.	.	.	G01 vs G03	0.7964
		T02	.	.	.	.	G01 vs G04	0.7964
		T02	.	.	.	.	G02 vs G03	0.7964

		T02	.	.	.	.	G02 vs G04	0.7964
		T02	.	.	.	.	G03 vs G04	0.7964
	Ruminococcaceae	T02	9.557 (2.923)	7.177 (2.446)	21.797 (2.923)	14.593 (2.578)	G01 vs G02	0.5343
		T02	.	.	.	.	G01 vs G03	0.0124 (*)
		T02	.	.	.	.	G01 vs G04	0.2405
		T02	.	.	.	.	G02 vs G03	0.0016 (**)
		T02	.	.	.	.	G02 vs G04	0.0808
		T02	.	.	.	.	G03 vs G04	0.1029
	Staphylococcaceae	T02	0.006 (0.297)	0.003 (0.249)	0.004 (0.297)	0.003 (0.262)	G01 vs G02	0.9993
		T02	.	.	.	.	G01 vs G03	0.9993
		T02	.	.	.	.	G01 vs G04	0.9993
		T02	.	.	.	.	G02 vs G03	0.9993
		T02	.	.	.	.	G02 vs G04	0.9993
		T02	.	.	.	.	G03 vs G04	0.9993
	Streptococcaceae	T02	7.185 (1.438)	5.243 (1.203)	0.914 (1.438)	1.747 (1.268)	G01 vs G02	0.3646
		T02	.	.	.	.	G01 vs G03	0.0174 (*)
		T02	.	.	.	.	G01 vs G04	0.0177 (*)
		T02	.	.	.	.	G02 vs G03	0.0476 (*)
		T02	.	.	.	.	G02 vs G04	0.0739
		T02	.	.	.	.	G03 vs G04	0.6652
Proteobacteria	Caulobacteraceae	T02	0.045 (0.038)	0.028 (0.032)	0.012 (0.038)	0.028 (0.034)	G01 vs G02	0.9046
		T02	.	.	.	.	G01 vs G03	0.9046
		T02	.	.	.	.	G01 vs G04	0.9046
		T02	.	.	.	.	G02 vs G03	0.9046
		T02	.	.	.	.	G02 vs G04	0.9991
		T02	.	.	.	.	G03 vs G04	0.9046
	Enterobacteriaceae	T02	1.054 (1.253)	6.886 (1.049)	0.945 (1.253)	0.485 (1.105)	G01 vs G02	0.0013 (**)
		T02	.	.	.	.	G01 vs G03	0.9512
		T02	.	.	.	.	G01 vs G04	0.9411
		T02	.	.	.	.	G02 vs G03	0.0013 (**)
		T02	.	.	.	.	G02 vs G04	0.0004 (***)
		T02	.	.	.	.	G03 vs G04	0.9411
	Oxalobacteraceae	T02	0.038 (0.020)	0.013 (0.017)	0.007 (0.020)	0.021 (0.018)	G01 vs G02	0.8400
		T02	.	.	.	.	G01 vs G03	0.8400

		T02	.	.	.	.	G01 vs G04	0.8400
		T02	.	.	.	.	G02 vs G03	0.8400
		T02	.	.	.	.	G02 vs G04	0.8400
		T02	.	.	.	.	G03 vs G04	0.8400
	Pseudomonadaceae	T02	0.259 (0.112)	0.087 (0.094)	0.050 (0.112)	0.118 (0.099)	G01 vs G02	0.7003
		T02	.	.	.	.	G01 vs G03	0.7003
		T02	.	.	.	.	G01 vs G04	0.7003
		T02	.	.	.	.	G02 vs G03	0.8192
		T02	.	.	.	.	G02 vs G04	0.8192
		T02	.	.	.	.	G03 vs G04	0.8192
Saccharibacteria	Unknown_Family	T02	10.806 (4.177)	12.139 (3.495)	23.556 (4.177)	16.947 (3.684)	G01 vs G02	0.8073
		T02	.	.	.	.	G01 vs G03	0.1203
		T02	.	.	.	.	G01 vs G04	0.4115
		T02	.	.	.	.	G02 vs G03	0.1203
		T02	.	.	.	.	G02 vs G04	0.4168
		T02	.	.	.	.	G03 vs G04	0.4115
-	unclassified	T02	0.688 (0.136)	0.855 (0.114)	0.389 (0.136)	0.525 (0.120)	G01 vs G02	0.4475
		T02	.	.	.	.	G01 vs G03	0.2530
		T02	.	.	.	.	G01 vs G04	0.4475
		T02	.	.	.	.	G02 vs G03	0.0642
		T02	.	.	.	.	G02 vs G04	0.1494
		T02	.	.	.	.	G03 vs G04	0.4600
